# Examining the influence of body fat distribution on standing balance and functional performance in overweight female patients with degenerative lumbar disease

**DOI:** 10.3389/fbioe.2024.1375627

**Published:** 2024-06-21

**Authors:** Jung-Hsuan Chen, Po-Jung Chen, Phunsuk Kantha, Yi-Ching Tsai, Dar-Ming Lai, Wei-Li Hsu

**Affiliations:** ^1^ School and Graduate Institute of Physical Therapy, College of Medicine, National Taiwan University, Taipei, Taiwan; ^2^ Faculty of Physical Therapy, Mahidol University, Nakhon Pathom, Thailand; ^3^ Division of Neurosurgery, Department of Surgery, National Taiwan University Hospital, Taipei, Taiwan; ^4^ Physical Therapy Centre, National Taiwan University Hospital, Taipei, Taiwan

**Keywords:** degenerative lumbar disease (DLD), overweight, standing balance, body fat distribution, center of pressure (COP), functional assessment, spinal alignment

## Abstract

**Introduction:** Degenerative lumbar disease (DLD) is a prevalent disorder that predominantly affects the elderly population, especially female. Extensive research has demonstrated that overweight individuals (categorized by body fat distribution) have a higher susceptibility to developing DLD and an increased risk of falling. However, there is limited research available on the standing balance and functional performance of overweight females with DLD.

**Aims:** To determine the impact of body fat distribution on standing balance and functional performance in overweight females with DLD.

**Methods:** This cross-sectional study evaluated thirty females with DLD were categorized into three types of body fat distribution based on body mass index (BMI) and waist-hip ratio, specifically as android-type, gynoid-type, and normal weight groups. In addition, a control group of ten age-matched females with normal weight was recruited. The Visual Analogue Scale, Roland Morris Disability Questionnaire, Cobb angle (Determined using x-ray), and body composition (Determined using the InBody S10), were conducted only on the DLD groups. All participants were assessed standing balance in the anteroposterior and mediolateral directions. The functional assessments included timed-up-and-go and 5-times-sit-to-stand tests.

**Results:** There were 10 people in each group. Android-type (Age = 65.00 ± 6.34 years; BMI = 26.87 ± 2.05 kg/m^2^), Gynoid-type (Age = 65.60 ± 4.99 years; BMI = 26.60 ± 1.75 kg/m^2^), Normal weight (Age = 65.70 ± 5.92 years; BMI = 22.35 ± 1.26 kg/m^2^), and Control (Age = 65.00 ± 5.23 years; BMI = 22.60 ± 1.12 kg/m^2^). The android-type group had higher body fat, visceral fat, and lower muscle mass (*p* < 0.05), along with an increased Cobb angle (*p* < 0.05). They showed greater ellipse area, total excursion, and mean distance in the anteroposterior direction (*p* < 0.05). During the functional performance assessments, the android-type group had longer durations in both the 5-times-sit-to-stand and timed-up-and-go tasks (*p* < 0.05).

**Conclusion:** Our study found that android-type overweight individuals showed postural instability, reduced functional performance, and insufficient lower limb muscle strength and mass. These findings might help physical therapists in planning interventions, as they imply that patients with DLD may require specific types of standing balance training and lower extremities muscle-strengthening based on their body fat distribution.

**Clinical Trial Registration:** ClinicalTrials.gov, identifier NCT05375201

## Introduction

Degenerative lumbar disease (DLD) is a common disorder in the elderly, especially in females, and it is characterized by degenerative alterations at multiple levels of the lumbar spine, the degeneration may lead to musculoskeletal changes (Stromqvist et al., 2013; Ravindra et al., 2018; Wang et al., 2022). The prevalence rates of both males and females have been increasing annually, with a similar trend, although females generally exhibit higher prevalence rates in the diagnosis of the vast majority of lumbar spine diseases compared to males (Parenteau et al., 2021). The cost of DLD on the healthcare system, with the surgical cohort averaging $50.84 per patient per month, compared to $29.34 per patient per month for the nonsurgical cohort (Kim et al., 2021). DLD can be diagnosed through medical imaging, such as X-ray or magnetic resonance imaging (Steurer et al., 2011; Hasz, 2012). DLD is classified into three sub-types: spinal stenosis, spondylolisthesis, and disc degeneration (Hussain et al., 2017; Parenteau et al., 2021; Wan et al., 2022). Low back pain, sensory deficits, claudication, and poor standing balance performance are common symptoms of DLD (Ravindra et al., 2018). Nowadays, with the development of medical care and the increase in average life expectancy, patients with DLD have become one of the main populations with a high demand for medical care (Stromqvist et al., 2013; Ravindra et al., 2018).

Overweight is associated with many common health conditions, such as joint degeneration, increased pain, and poor daily activity performance (Pataky et al., 2014; Chen et al., 2021; Goode et al., 2022). Previous studies have reported that overweight is one of the main contributing factors of DLD (Hussain et al., 2017; Parenteau et al., 2021). Furthermore, body fat distribution may result in different structural changes that affect the loading of joints and the alignment of spine segments (Romero-Vargas et al., 2013). The increase in visceral fat ratio was positively correlated with the lumbar lordosis curve, leading to changes in spinal alignment (Taspinar, 2017). The prevalences of low back pain and DLD rise as body mass index (BMI) increases (Heuch et al., 2013; Smuck et al., 2014; Sheng et al., 2017). BMI greater than 25 kg/m^2^ increases the risk of lumbar degeneration (Liuke et al., 2005; Onyemaechi et al., 2016; Alangari et al., 2022).

Individuals with overweight are further classified based on different types of body fat distribution, determined by their waist–hip ratio (WHR). Android-type overweight is defined as a body mass index (BMI) greater than 25 kg/m^2^ and WHR greater than 0.85 (World Health Organization, 2008). Android-type overweight is also known as central type overweight, in which the fat accumulates in the abdominal region (Janjic, 1996). Gynoid-type overweight is defined as a BMI greater than 25 kg/m^2^ and WHR less than 0.85 (World Health Organization, 2008). Gynoid-type overweight is a female-domain body fat distribution, which means the fat accumulates mainly on the hips or lower extremities (Menegoni et al., 2009; Hita-Contreras et al., 2013; Tchernof and Despres, 2013). Overweight and body fat distribution in the elderly affect not only general health but also functional performance and the safety of daily activities (G. R. Neri S. et al., 2020).

Standing balance is defined as the ability to maintain and stabilize balance while performing any activity in upright posture (Winter et al., 1996). The function of standing balance is to reduce postural sway, avoid postural instability, and decrease falls (Pollock et al., 2000). Moreover, DLD may cause changes in the biomechanical structure, neurological dysfunction, pain, and alteration of the balance strategy, resulting in a high risk of falling and poor functional performance (Ravindra et al., 2018; Wong et al., 2019).

The effects of DLD on standing balance can be attributed to the following four factors: (1) Changes in sagittal alignment: DLD can cause changes in sagittal alignment, such as a forward shift in the lumbar spine. The imbalance in sagittal alignment, resulting in a forward shift of the center of mass, may affect standing balance. (Barrey et al., 2011; Ito et al., 2020); (2) Increased low back pain and disability: DLD is commonly associated with low back pain, which can lead to pain avoidance behaviors and increased trunk muscle co-activation (Ravindra et al., 2018). These adaptations in balance strategy may result in a more rigid stance (Brumagne et al., 2008; Ito et al., 2020). Moreover, reduced daily activities due to increased pain intensity can contribute to disability and impaired upright balance (Ravindra et al., 2018; Wong et al., 2019); (3) Alterations in proprioception: Elderly individuals with DLD often experience alterations in their musculoskeletal, sensory, and proprioception systems, leading to balance instability (Kanekar and Aruin, 2014; Wong et al., 2019). As environmental interference increases, individuals with DLD struggle to maintain efficient standing balance, thereby increasing their risk of falling (Ravindra et al., 2018; Wong et al., 2019); and (4) Muscle weakness: Individuals with DLD often exhibit core instability, lower extremity weakness, and decreased physical activity, all of which can impair standing balance control (Ammendolia, 2014; Ravindra et al., 2018).

The effects of overweight on standing balance can be attributed to the following four factors: (1) Changes in spinal alignment: Excess weight and fat mass can lead to changes in spinal alignment, such as increased lumbar lordosis curve and pelvic anterior tilt (Ando et al., 2020; Buckland et al., 2020). These changes can result in increased pressure on the lumbar spine and alterations in alignment (Ando et al., 2020; Buckland et al., 2020). The android-type fat distribution is particularly associated with noticeable changes in spinal alignment and the severity of lumbar degeneration (Hirjakova et al., 2018; Ando et al., 2020); (2) Increased inflammatory factors: The body’s excess adipose tissue in overweight individuals can cause chronic low-grade inflammation. This inflammation can raise pain levels and decrease the efficiency of self-recovery (Hussain et al., 2017; Kawai et al., 2021); (3) Decreased plantar sensitivity: A prolonged burden of excess weight on the feet can lead to a decrease in plantar sensitivity (Wu and Madigan, 2014). Plantar sensitivity refers to the ability of the plantar mechanoreceptors on the feet to detect pressure and deformation in the skin (Andreato et al., 2020). Impairment of these mechanoreceptors and a decrease in plantar sensitivity can contribute to a decrease in standing stability (Winter et al., 1990; Wu and Madigan, 2014); and (4) Increased gravitational torque: The extra fat mass in the abdominal region shifts the center of mass (CoM) forward, increasing the torque of gravity and altering body orientation (Wu and Madigan, 2014; Son, 2016). Therefore, individuals who are overweight may struggle to maintain their standing balance, leading to a higher risk of falling and poorer functional performance (Lee et al., 2020).

Individuals with DLD or overweight may experience worsened standing balance and poor functional performance (Iversen et al., 2009; Hita-Contreras et al., 2013; Truszczynska et al., 2014). However, previous studies have not compared standing balance and functional performance in individuals with DLD among the android-type, gynoid-type, and normal weight groups. Therefore, this study aimed to compare standing balance and functional performance in female DLD patients among various types of body fat distribution.

## Material and methods

### Study design

This cross-sectional study was designed to evaluate pain intensity, disability, spinal alignment, body composition, standing balance, and functional activities performance in female individuals with DLD among normal weight, android-type, and gynoid-type groups. This study was approved by the Research Ethics Committee of National Taiwan University Hospital (IRB reference number 202003149RINC), and the trial was registered at ClinicalTrials.gov (identifier NCT05375201).

### Participants

The sample size estimation was calculated using a significance level of 0.05, a power of 0.8, and an effect size set at 0.8. The sample size for each group in this study was determined to be 10. The participants in the DLD group were eligible for participation if they had to meet the following inclusion criteria: (1) female, aged 50–80 years; (2) capable of standing and walking independently without assistance; and (3) diagnosed with DLD based on X-ray imaging examination. The exclusion criteria were as follows: (1) a history of spine surgery or lower extremity surgery; (2) neurological disorders such as spinal cord injury or stroke; (3) diabetes or vestibular disease, which may impair proprioception and cause balance problems; and (4) ankylosing spondylitis or rheumatoid arthritis. Patients with DLD were allocated into three groups based on body fat distribution: the normal weight group, android-type group, and gynoid-type group. The measurement of body fat distribution types was conducted using the WHR. Participants were instructed to stand upright and remain still while a measuring tape was used to measure the circumference of their waist (along the middle line from the lowest rib to the iliac crest) and hips (at the widest portion of the buttocks) (World Health Organization, 2008).

The participants in the age-matched healthy controls (control group) were eligible for participation if they had (1) female, aged 50–80 years; (2) capable of standing and walking independently without assistance; (3) normal BMI; and (4) no neck and back pain, musculoskeletal injury of the lower extremities or spine, vestibular dysfunction, or other neurological dysfunction. The exclusion criteria for the control group were the same as those for the DLD group. The control group was.

### Study procedures

**FIGURE 1:**
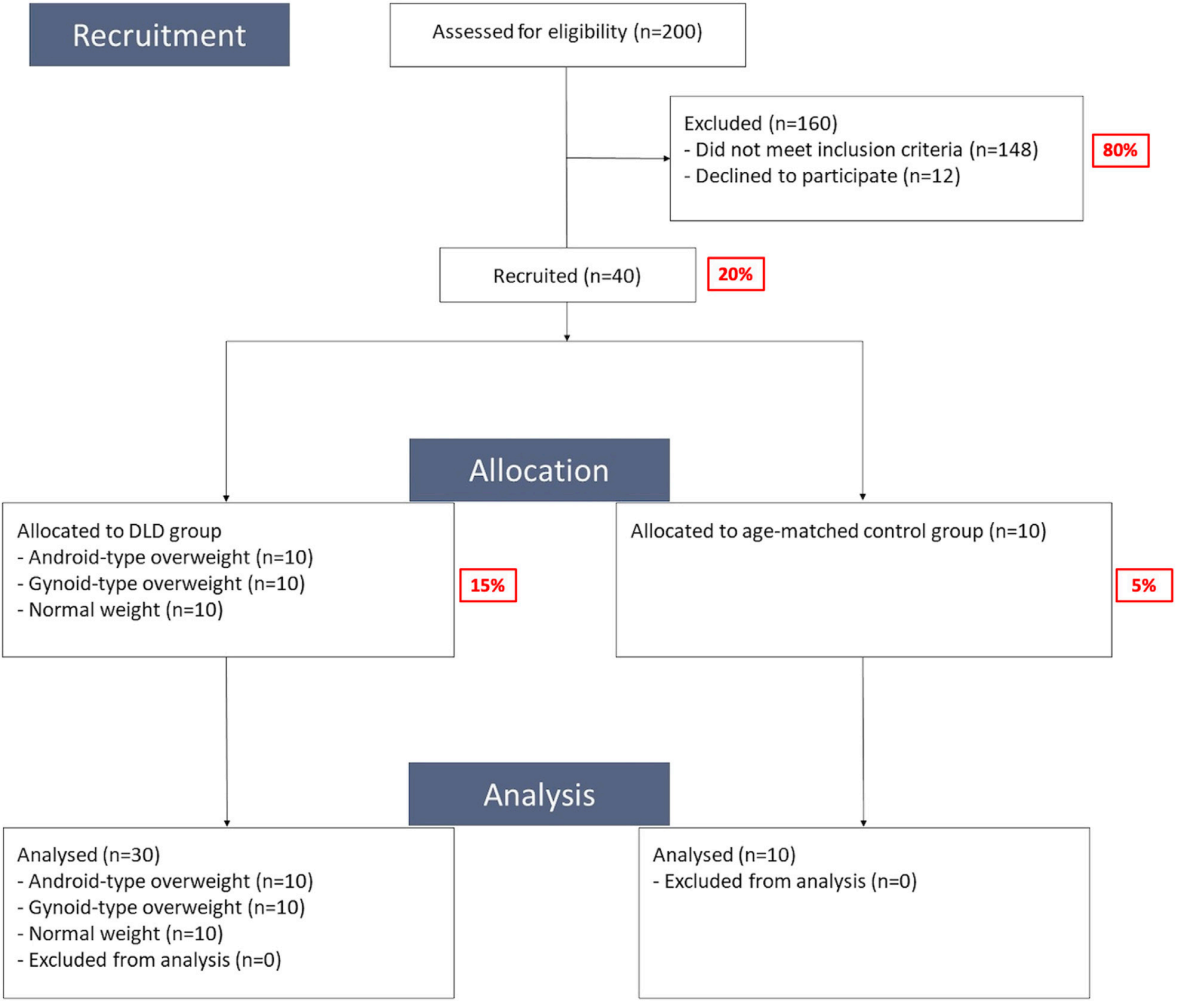
Flowchart of study procedures.

The flow chart of this study is shown in Figure 1. All of the participants received an explanation of the experiment and signed an informed consent form at the clinic of National Taiwan University Hospital. Data collection for all participants encompassed demographic information and clinical outcomes. Demographic information included age, body height, body weight, BMI, WHR, and spinal alignment. Measurement of WHR is performed by physiotherapists trained through a standardized training program. Additionally, questionnaires were used to gather information on pain intensity, disability index. Clinical outcomes included measurements of body composition, standing balance, and functional performance assessments.

### Study measures

#### Clinical questionnaires and self-reported outcome measures

The clinical questionnaires were conducted in the DLD group only. The following clinical questionnaires were collected: (1) the Visual Analog Scale (VAS) was used to measure the intensity of low back pain as perceived by participants (Chiarotto et al., 2019). The VAS is a highly valid tool for evaluating acute pain (95% confidence interval, 0.96–0.98), and it is commonly used in clinical pain assessment (Bijur et al., 2001). The VAS is a self-assessed score ranging from 0 to 10, with higher scores indicating higher pain intensity. Scores of 0 indicate no pain, scores of one to three indicate mild pain, scores of 4–6 indicate moderate pain, and scores of 7–10 indicate severe pain (Chiarotto et al., 2019); and (2) The Roland–Morris Disability Questionnaire (RMDQ) is a 24-item scale used to measure the score of pain-related disability (Roland and Morris, 1983). The test–retest reliability of the RMDQ is 0.94, with a 95% confidence interval of 0.90–0.97 (Yi et al., 2012). Higher scores indicate higher levels of pain-related disability. Scores of 0 indicate no disability, scores of 1–8 indicate mild disability, scores of 9–16 indicate moderate disability, and scores of 17–24 indicate severe disability (Roland and Morris, 1983).

#### Spinal alignment

The spinal alignment assessment was conducted in the DLD group only. The Cobb angle from the X-ray imaging examination was used to evaluate spinal alignment. The Cobb angle was analyzed using a customized MATLAB program (The MathWorks, Natick, MA, United States). The definition of the Cobb angle for lumbar lordosis is the angle formed by the intersection point of perpendiculars drawn to the parallel lines between the L1 vertebra and the sacral plate (Sparrey et al., 2014). The intraclass correlation coefficient for Cobb angle measurement was 0.92, with a 95% confidence interval of 0.92–0.97 (Tanure et al., 2010). Measurement of Cobb angle is performed by physiotherapists trained through a standardized training program.

#### Body composition measurement

Body composition measurement was conducted in the DLD group only. Body composition was measured using the Inbody S10 (Biospace, Seoul, Korea). The parameters of body composition included body fat percentage, visceral fat area, lean muscle mass of trunk, and average muscle mass of the lower extremities. Participants were asked to sit quietly for 10 min prior to the measurement, keeping their backs upright, arms resting at their sides, and thighs not touching each other. Electrodes were placed on both ankles, middle fingers, and thumbs.

#### Standing balance assessment

##### Procedure of standing balance

**FIGURE 2:**
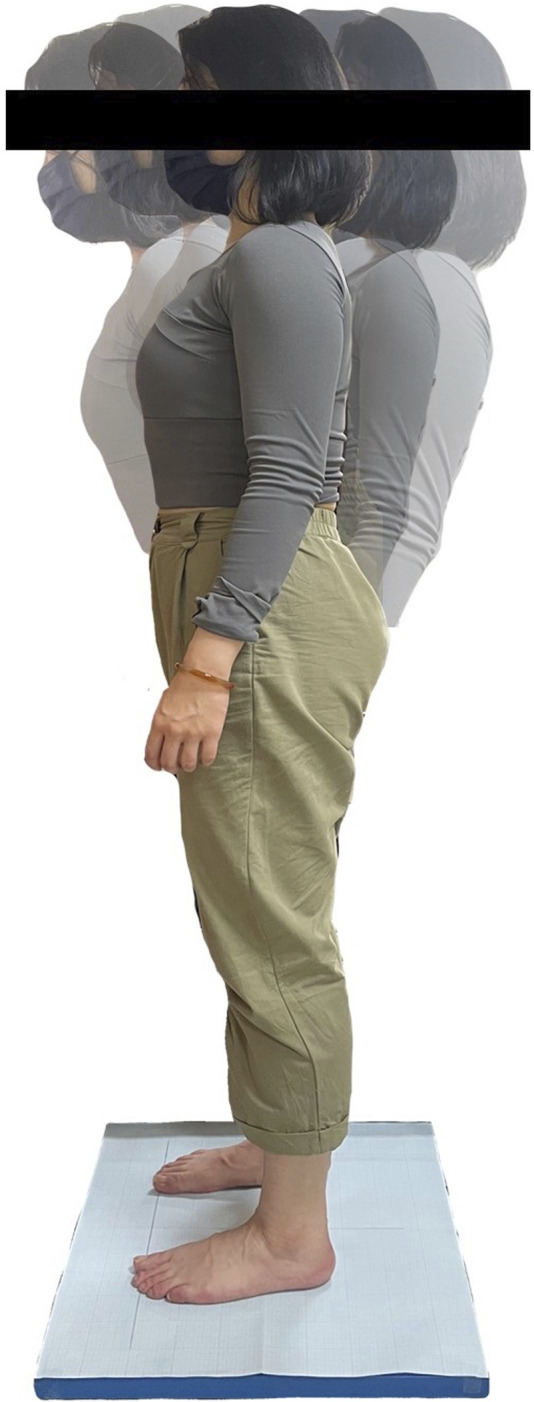
Standing balance assessment setup.

All participants were asked to perform quiet standing for 35 s with their eyes open. Participants stood barefoot with their arms by their sides and their feet shoulder-width apart (Figure 2), and footprint paper was used to record the stance width. A force plate (Kistler 9286A, Kistler Instrument AG, Winterthur, Switzerland) was used to estimate the center of pressure (CoP) during the standing balance assessment.

#### Processing center of pressure (CoP)

Center of pressure (CoP) is the location of the ground reaction vector from a force plate, which is equal to and opposite to a weight average of all downward force. In clinical application, CoP variables is commonly used to assess and quantify standing balance (Winter et al., 1996). The parameters used to assess standing balance were as follows: 95% confidence ellipse area, total excursion, mean distance, and mean frequency. (1) 95% confidence ellipse area: measuring the total area of the CoP trajectory covered by the AP and ML directions. The smaller the area, the better the standing balance performance (2) Total excursion: the total length of the CoP path during testing time. The lower the total excursion, the better the standing balance performance; (3) Mean distance: measuring how far the sway is from the center of the force plate. The smaller the CoP mean distance, the better the standing balance performance; and (4) Mean frequency: measuring how much the sway vibrates. The lower the CoP mean frequency, the better the standing balance performance (Quijoux et al., 2021).

The CoP analog data set was sampled at a rate of 1,000 Hz using a customized LabVIEW program (National Instruments, Austin, TX, United States) to calculate the CoP based on the ground reaction force and moment in the anterior–posterior (AP) and medial–lateral (ML) directions. The CoP data were filtered and processed using a fourth-order Butterworth low-pass filter with a cutoff frequency of 10 Hz using MATLAB R2021a software (MathWorks, Natick, MA, United States) (Quijoux et al., 2021).

#### Functional performance

The functional performance assessments were the five-times-sit-to-stand test (5STS) and the timed-up-and-go test (TUG) for all participants.

The 5STS is used to evaluate muscle strength in the lower extremities and predict the risk of falling (Lord et al., 2002; Goldberg et al., 2012). In the starting position, participants sit against the back of the chair (45 cm height) with their arms crossed. After the timer is started, participants are asked to repeatedly stand up fully and sit down five times, maintaining an upright trunk with extended hips and knees. Researchers use a digital stopwatch to record the time. A longer duration indicates lower muscle strength of the lower extremities and a higher risk of falling. According to previous studies, the 5STS cut-off point indicating a higher risk of falling is > 14 s and that for lower muscle strength of the lower extremities is > 16 s (Goldberg et al., 2012).

The TUG test is used to assess participants' risk of falling and their walking ability (Bohannon, 2006; Kamide et al., 2011). Participants are instructed to sit in the chair (45 cm height) as the starting position. In response to a cue, participants must stand up, walk 3 m, turn around, walk back to the chair, and sit down. Researchers use a digital stopwatch to record the time. The TUG test was performed twice, and the average value was taken. A longer duration indicates a higher risk of falling and poorer walking ability. According to previous studies, the TUG cut-off point of higher risk of falling is >12 s (Bohannon, 2006; Kamide et al., 2011).

#### Statistical analysis

The descriptive data of the participants are presented as means and standard deviations for continuous data. One-way ANOVA was used to compare the differences in pain intensity, disability, body composition, and spinal alignment among female DLD patients with normal weight, android-type overweight, and gynoid-type overweight. Age-matched healthy controls were included in the comparisons for standing balance and functional performance. The significance level was set at 0.05, and *post hoc* analysis was conducted using the Scheffé test. The statistical analysis was performed in PASW Statistics 25 software for Windows (SPSS, Chicago, IL, United States).

## Results

A total of 40 participants were recruited for this study (DLD group, *n* = 30; age-matched control group, *n* = 10). The demographic characteristics of the participants are summarized in Table 1. There were no significant differences in age and height among three types of DLD participants and control group. However, the weight and BMI of Android-type and Gynoid-type DLD groups were significantly higher than those of the normal weight DLD or control groups.

**TABLE 1:** Demographics characteristics of DLD participants.

Characteristics	Android-type (n = 10)	Gynoid-type (n = 10)	Normal weight (n = 10)	Control group (n = 10)	*p*-value
Age (years)	65.00 ± 6.34	65.60 ± 4.99	65.70 ± 5.92	65.00 ± 5.23	*p* = 0.99
Height (cm)	156.94 ± 5.58	156.18 ± 2.68	156.18 ± 5.50	156.00 ± 2.50	*p* = 0.76
Weight (kg)	66.27 ± 7.41	64.50 ± 4.22	54.55 ± 4.25	55.30 ± 3.65	*p* < 0.01
BMI (kg/m^2^)	26.87 ± 2.05	26.60 ± 1.75	22.35 ± 1.26	22.60 ± 1.12	*p* < 0.01

Values are mean ± SD, or number.

BMI: body mass index.

### Pain intensity and disability

The results of pain intensity (VAS) and disability (RMDQ) among female DLD patients in the normal weight, android-type overweight, and gynoid-type overweight groups are shown in Table 2. The VAS scores showed no significant differences among the normal weight group, android-type overweight group, and gynoid-type overweight group (*F* (2,27) = 0.547, *p* = 0.585). Similarly, the RMDQ scores showed no significant differences among the normal weight group, android-type overweight group, and gynoid-type overweight group (*F* (2,27) = 0.543, *p* = 0.587).

**TABLE 2:** Results of clinical questionnaires of DLD group.

	Android-type (n = 10)	Gynoid-type (n = 10)	Normal weight (n = 10)	*p*-value
VAS	5.50 ± 1.58	5.30 ± 1.88	6.20 ± 2.48	*p* = 0.94
RMDQ	11.90 ± 2.92	11.40 ± 6.51	13.70 ± 5.45	*p* = 0.56

Values are mean ± SD, or number.

VAS: visual analogue scale; RMDQ: Roland-Morris disability questionnaire.

### Body composition

**FIGURE 3:**
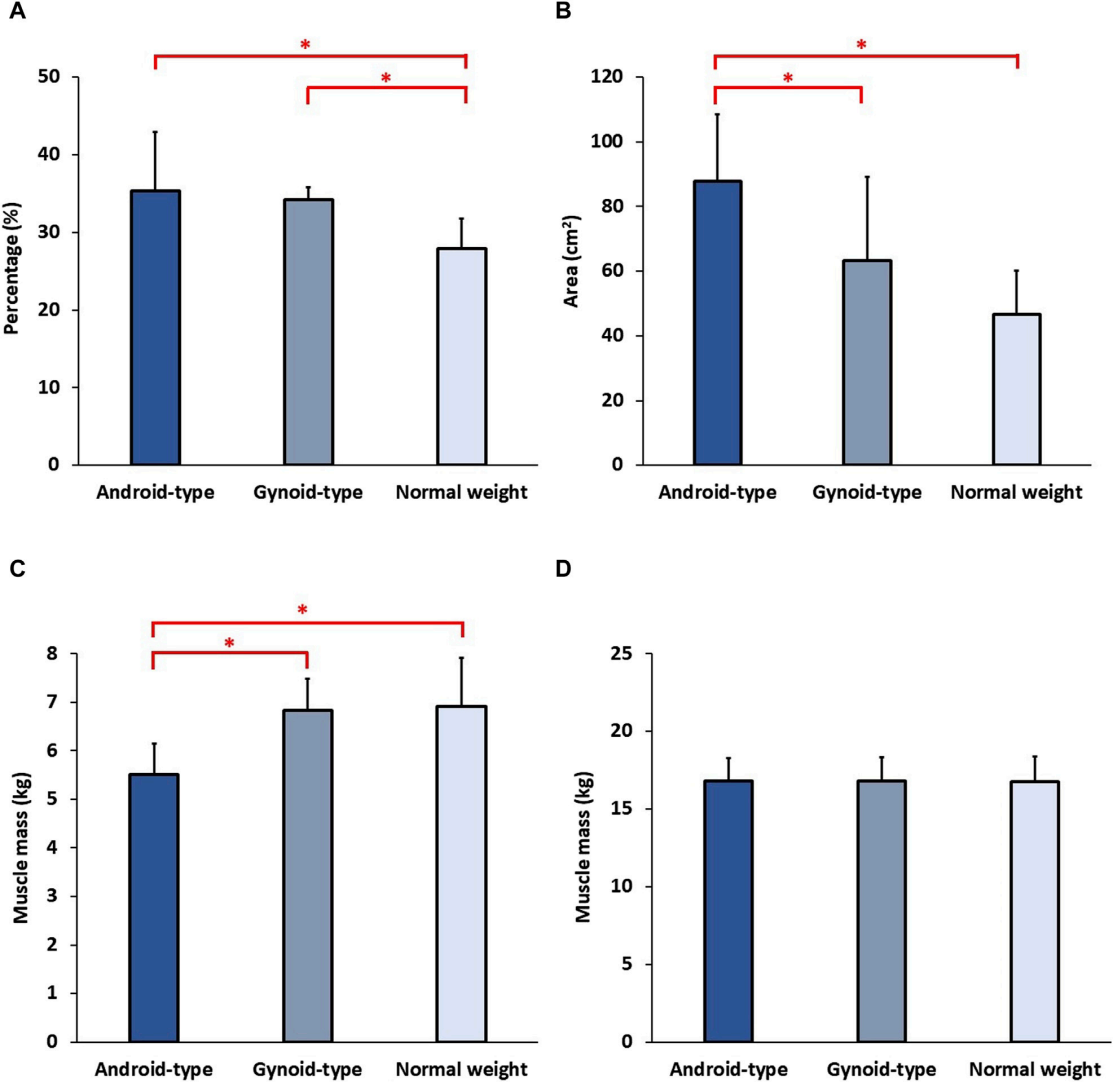
Body composition among the three DLD groups. **(A)** Body fat percentage; **(B)** Visceral fat area; **(C)** Average lean muscle mass of lower extremities; **(D)** Lean muscle mass of trunk. * Indicates significant differences among the DLD groups.

The detailed results of body composition among female DLD patients in the normal weight, android-type overweight, and gynoid-type overweight groups are shown in Figure 3. There was a statistically significant difference in body fat percentage among the DLD groups (*F* (2,27) = 8.401, *p* = 0.001). Specifically, both the android-type overweight group (mean = 34.61 ± 5.33%, *p* = 0.001) and the gynoid-type overweight group (mean = 33.97 ± 4.44%, *p* = 0.013) exhibited significantly higher body fat percentages compared with the normal weight group (mean = 25.92 ± 4.09%). Additionally, there was also a statistically significant difference in visceral fat area among the DLD groups (*F* (2,27) = 9.378, *p* = 0.001). The android-type overweight group had a significantly larger visceral fat area (mean = 84.50 ± 20.70 cm^2^) than those of the normal weight group (mean = 45.50 ± 15.00 cm^2^, *p* = 0.001) and the gynoid-type overweight group (mean = 58.90 ± 24.70 cm^2^, *p* = 0.021). However, no significant differences were observed between the gynoid-type overweight group and the normal weight group. The lean muscle mass of trunk was no significant difference among the three DLD groups.

### Spinal alignment

**FIGURE 4:**
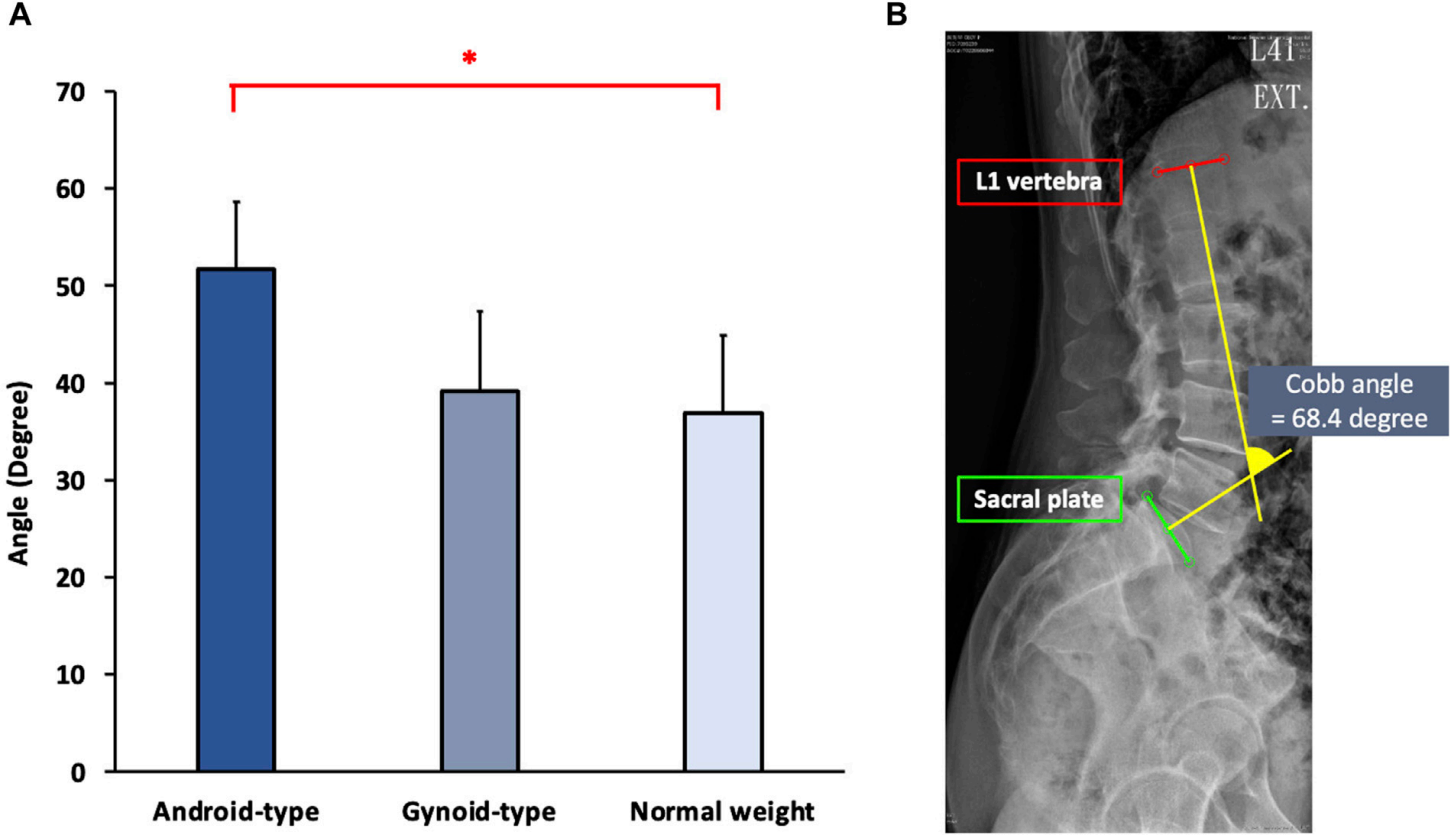
Spinal alignment among the three DLD groups. **(A)** Cobb angle; **(B)** The Cobb angle in one representative subject. * Indicates significant differences among the DLD groups.

The results of spinal alignment among female DLD patients in the normal weight, android-type overweight, and gynoid-type overweight groups are shown in Figure 4.

Significant differences in spinal alignment were observed among the DLD groups (*F* (2,27) = 9.945, *p* = 0.001). The android-type overweight group exhibited a significantly increased Cobb angle (mean = 50.42 ± 6.73°) compared with that of the normal weight group (mean = 36.61 ± 8.00°) (*p* = 0.001). However, there were no significant differences between the gynoid-type overweight group (mean = 39.26 ± 7.25°) and the normal weight group (mean = 36.61 ± 8.00°).

### Standing balance

**FIGURE 5:**
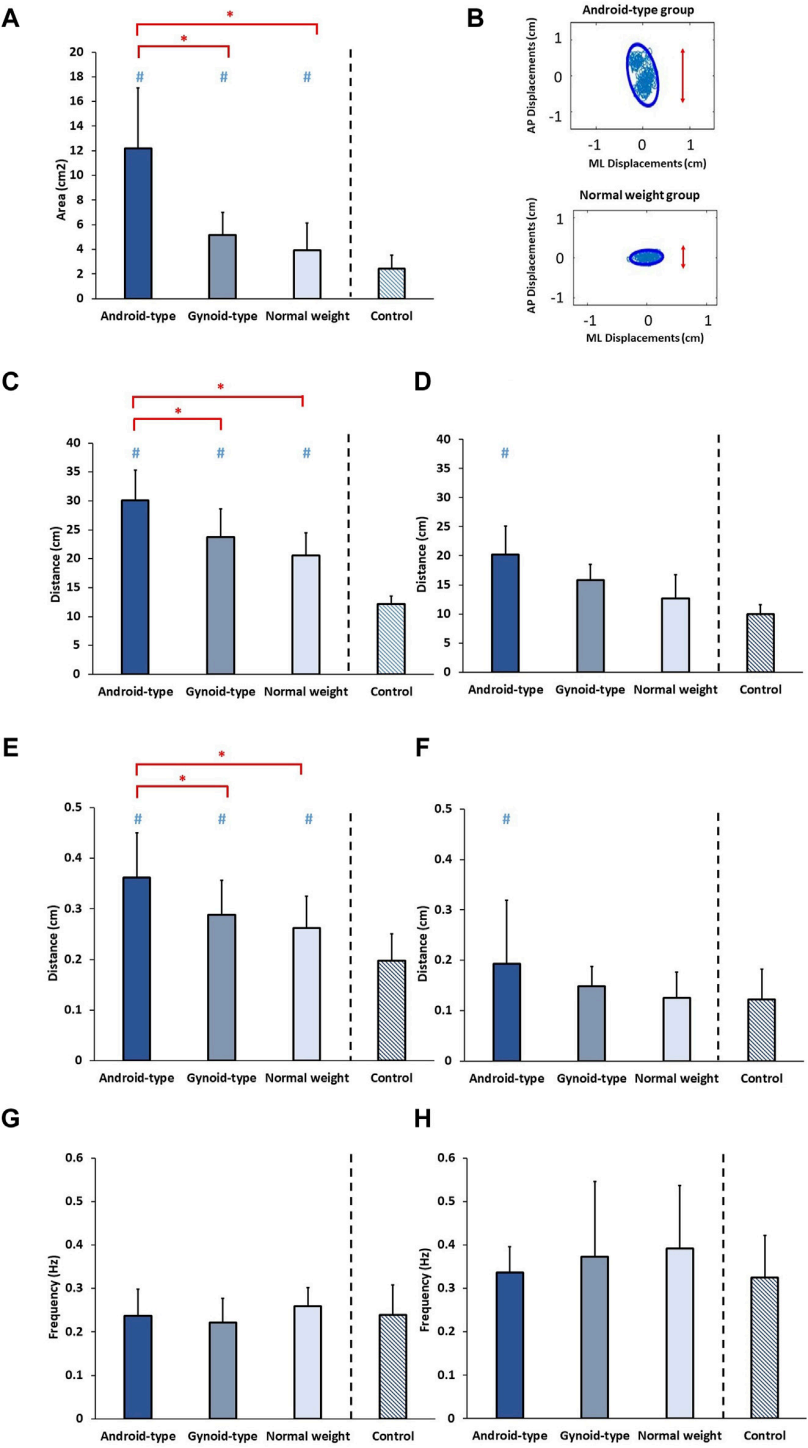
Standing balance among the three DLD groups and age-matched control group. **(A)** 95% Confidence ellipse area; **(B)** 95% Confidence ellipse area in representative subjects; **(C)** Total excursion in AP direction; **(D)** Total excursion in ML direction; **(E)** Mean distance in AP direction; **(F)** Mean distance in ML direction; **(G)** Mean frequency in AP direction; **(H)** Mean frequency in ML direction. * Indicates significant differences among the DLD groups. # Indicates significant differences in comparison to the aged-matched control group. AP: Anterior-posterior; ML: Medial-lateral.

The results of standing balance among the three DLD groups and age-matched control group are shown in Figure 5.

#### 95% confidence ellipse area

A statistically significant difference in the 95% confidence ellipse area was observed among the DLD groups and the age-matched control group (*F* (3,34) = 23.127, *p* = 0.001). In the DLD groups, the android-type overweight group exhibited a significantly increased 95% confidence ellipse area (mean = 12.06 ± 4.35 cm^2^) compared with those of the normal weight group (mean = 4.20 ± 2.16 cm^2^) (*p* = 0.012) and the gynoid-type group (mean = 4.91 ± 1.83 cm^2^) (*p* = 0.037). However, no significant differences were observed between the gynoid-type overweight and normal weight groups. Furthermore, the healthy controls exhibited a smaller 95% confidence ellipse area (mean = 2.41 ± 1.12 cm^2^) than those of the normal weight group, the android-type overweight group, and the gynoid-type overweight group.

#### Total excursion

A significant difference in the total excursion in the AP direction was observed among the DLD groups and the age-matched control group (*F* (3,34) = 33.454, *p* = 0.001). In the DLD groups, the android-type overweight group displayed a significantly greater total excursion in the AP direction (mean = 31.18 ± 5.28 cm) compared with those of the normal weight group (mean = 20.37 ± 3.85 cm) (*p* = 0.021) and the gynoid-type group (mean = 23.81 ± 4.30 cm) (*p* = 0.032). However, no significant differences were found between the gynoid-type overweight and normal weight groups. Additionally, healthy controls exhibited a smaller total excursion (mean = 12.19 ± 1.33 cm) than those of the normal weight group, the android-type overweight group, and the gynoid-type overweight group.

A significant difference in the total excursion in the ML direction was observed between the DLD groups and the age-matched control group (*F* (3,34) = 18.122, *p* = 0.001). However, in the DLD groups, there were no significant differences between the gynoid-type overweight group (mean = 16.25 ± 2.57 cm) and the normal weight group (mean = 11.98 ± 3.53 cm), nor between the android-type overweight group (mean = 20.16 ± 4.34 cm) and the normal weight group. Only the DLD group with android-type overweight exhibited a significantly greater total excursion than that of the healthy controls (mean = 9.95 ± 1.59 cm), while the other types of DLD (gynoid-type overweight and normal weight) did not differ significantly from healthy controls.

#### Mean distance

There was a significant difference in the mean distance in the AP direction among the DLD groups and the age-matched control group (*F* (3,34) = 8.068, *p* = 0.001). In the DLD groups, the android-type overweight group had a significantly greater mean distance in the AP direction (mean = 0.35 ± 0.08 cm) than those of the normal weight group (mean = 0.26 ± 0.06 cm) (*p* = 0.001) and the gynoid-type group (mean = 0.29 ± 0.06 cm) (*p* = 0.046). However, there were no significant differences between the gynoid-type overweight and normal weight. Additionally, healthy controls exhibited a shorter mean distance (mean = 0.19 ± 0.05 cm) than those of the normal weight group, the android-type overweight group, and the gynoid-type overweight group.

A significant difference in the mean distance in the ML direction was observed among the DLD groups and the age-matched control group (*F* (3,34) = 6.068, *p* = 0.014). However, in the DLD groups, there were no significant differences between the gynoid-type overweight group (mean = 0.14 ± 0.04 cm) and the normal weight group (mean = 0.14 ± 0.05 cm), nor between the android-type overweight group (mean = 0.20 ± 0.12 cm) and the normal weight group. Only the DLD group with android-type overweight exhibited a significantly greater mean distance than that of the healthy controls (mean = 0.12 ± 0.06 cm), while participants with the other types of DLD (gynoid-type overweight and normal weight) did not differ significantly from healthy controls.

#### Mean frequency

No significant differences were found in the mean frequency in the AP direction (*F* (3,34) = 0.413, *p* = 0.745) or in the ML direction (*F* (3,34) = 1.128, *p* = 0.351) among the DLD groups and the age-matched control group.

### Functional performance

**FIGURE 6:**
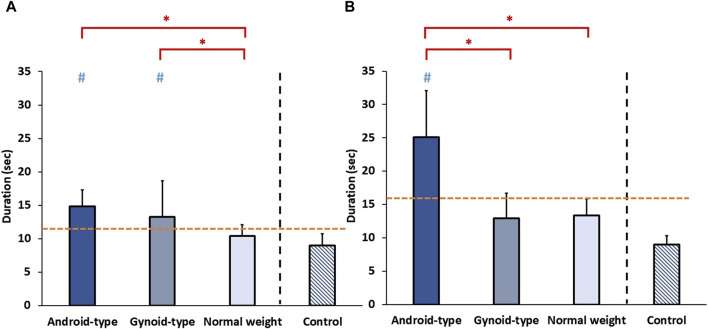
Functional performance among the three DLD groups and age-matched control group. **(A)** Timed-up-and-go test (TUG) **(B)** 5-times-sit-to-stand test (5STS). * Indicates significant differences among the DLD groups. # Indicates significant differences in comparison to the aged-matched control group. -- Indicates the cut-off points of **(A)** high falling risk and **(B)** low muscle strength of lower muscle extremities.

The results on functional performance among the three DLD groups and age-matched control group are shown in Figure 6.

#### Timed-up-and-go test (TUG)

A significant difference in the duration of TUG was observed among the DLD groups and the age-matched control group (*F* (3,34) = 5.748, *p* = 0.003). In the DLD groups, the duration of TUG was significantly longer in the android-type overweight (mean = 14.74 ± 2.23 s) (*p* = 0.015) and gynoid-type groups (mean = 12.57 ± 4.97 s) (*p* = 0.033) than in the normal weight group (mean = 10.53 ± 1.70 s). Additionally, healthy controls exhibited a shorter duration of TUG (mean = 9.00 ± 1.77 s) than those of the android-type overweight group, and the gynoid-type overweight group.

#### 5-Times-sit-to-stand test (5STS)

A significant difference in the duration of 5STS was observed among the DLD groups and the age-matched control group (*F* (3,34) = 13.145, *p* = 0.001). In the DLD groups, the duration of 5STS was significantly longer in the android-type overweight group (mean = 22.49 ± 8.24 s) than in the gynoid-type group (mean = 12.69 ± 3.36 s) (*p* = 0.033) and normal weight group (mean = 13.79 ± 2.65 s) (*p* = 0.017). Additionally, healthy controls exhibited a shorter duration of TUG (mean = 8.99 ± 1.32 s) than those of the android-type overweight group (*p* = 0.012) and the gynoid-type overweight group (*p* = 0.019). Only the DLD group with android-type overweight exhibited a significantly longer duration than that of the healthy controls (mean = 8.99 ± 1.32 s), while the other DLD groups (gynoid-type overweight and normal weight) did not differ significantly from the healthy control group.

## Discussion

**FIGURE 7:**
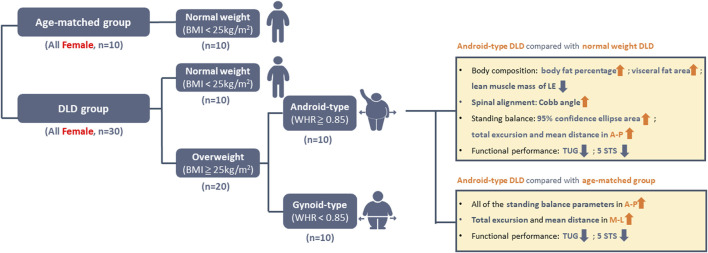
The main findings related to android-type DLD.

The key findings among individuals with DLD combined android-type in this study were as follows: (1) Higher body fat percentage and visceral fat percentage; (2) Increased lumbar lordosis curve, leading to a forward shift of CoM; (3) Inferior standing balance performance in the Anterior-posterior (AP) direction; and (4) Deficient lower extremity muscle strength and muscle mass based on functional performance tests and body composition measurements (Figure 7).

### Severity of pain and disability

In our study, there were no differences in the severity of pain and disability among individuals with DLD in the normal weight, android-type overweight, and gynoid-type overweight groups. Previous studies have indicated that excess adipose tissue in the body can lead to chronic low-grade inflammation, resulting in pain and increased disability (Hussain et al., 2017; Kawai et al, 2021; Zheng et al., 2022). However, some research has shown that a decrease in BMI may not be associated with the severity of back pain and disability after bariatric surgery or physiotherapy treatment (Mangwani et al., 2010; Koremans et al., 2021), which is consistent with our study. Pain is the subjective experience. In our suggestion, the subjective questionnaires used may not have been sufficiently sensitive to detect differences in pain severity among patients experiencing consistent pain. Thus, subjective questionnaires may not entirely reflect the intensity of pain and disability experienced by individuals with DLD. Chronic low back pain has multidimensional qualities, encompassing aspects beyond the physiological realm, including emotional and cognitive domains. For instance, anxiety and depression can exacerbate the perception of pain (Petrucci et al., 2021).

### Changes in spinal alignment

The android-type overweight group exhibited a higher body fat percentage and visceral fat area compared with the normal weight group in the current study. The increase in visceral fat ratio was positively correlated with the lumbar lordosis curve, leading to changes in spinal alignment (Taspinar, 2017). In our study, the android-type overweight group demonstrated a significantly greater lumbar Cobb angle than the normal weight group. These findings are consistent with previous research, which has shown that excessive body weight and adiposity in the abdominal region have a significant impact on the lumbar lordosis curve (Saludes et al., 2022). An increase in body fat percentage in the abdominal region may increase burden on the lumbar spine, shift the CoM forward and increase lumbar lordosis (Romero-Vargas et al., 2013; Sheng et al., 2017).

### Standing balance and functional performance among the three DLD groups

Among the three DLD groups, the android-type group demonstrated poorer standing balance in the AP direction but not in the ML direction. During quiet standing, individuals in the android-type group exhibited farther and wider postural sway in the AP direction than those of the normal weight and gynoid-type groups.

Our results might be explained by several factors that contribute to poor standing balance performance in the AP direction in the android-type overweight group. These factors include poor spinal alignment, a forward shift of the CoM, and insufficient lower extremity muscle strength. Each factor is illustrated as follows:

(1) Poor spinal alignment and forward shift of CoM: The android-type group had a large Cobb angle due to excess fat accumulation in the abdominal region, resulting in increased lumbar lordosis (Son, 2016; Sheng et al., 2017). This change in spinal alignment caused the CoM to shift forward and increased gravitational torque, making it more challenging to maintain standing balance (Son, 2016). The non-ideal alignment of the lumbar spine and the forward shift of the CoM may result in instability in standing balance in the AP direction.
(2) Insufficient lower extremities muscle strength in android-type: Previous studies have reported decreased functional performance in tests such as the TUG and 5STS in the android-type group, indicating inadequate lower muscle strength in the lower extremities (Lord et al., 2002; Bohannon, 2006; Kamide et al., 2011; Goldberg et al., 2012).

According to our study results, the average lean muscle mass of the lower extremities was lowest in the android-type group. Insufficient muscle strength in the lower extremities may make it more difficult to maintain upright standing balance (de Maio Nascimento et al., 2022).

### Standing balance and functional performance among DLD groups and the age-matched control group

Compared with the age-matched control group, all three types of DLD groups demonstrated poor standing performance in the AP direction. Additionally, the android-type group exhibited significantly greater sway area, total excursion, and mean distance in the ML direction compared with the age-matched control group. Previous research has indicated that excessive fat accumulation in the abdominal region can alter an individual’s body orientation which shifted the CoM forward during the maintenance of an upright posture (Porto et al., 2012). This change is typically compensated for by adopting a wide-base standing position (Porto et al., 2012).

However, in our study, we asked participants keep their feet shoulder-width apart and recorded their footprints. This position restricted the android-type group from adopting a wide-base standing position, thereby preventing them from compensating for the altered body orientation. Thus, the android-type group displayed poor standing balance in the ML direction.

Regarding functional performance, the overweight DLD groups (android-type and gynoid-type) exhibited a higher risk of falls compared with both the normal weight group and the age-matched control group. However, the increased falling risk and poor standing balance observed in the android-type group may be attributed to insufficient lower extremity muscle strength. For clinical implication, these findings indicate the high demand for lower extremity strength in the android-type group to improve their balance and reduce the risk of falls, which is consistent with previous research (Porto et al., 2012; Yadav et al., 2022).

### Study limitations

There were variations in the participants' lifestyles and symptom durations. The different types of neurogenic symptoms might have been influenced by the individual lifestyles or duration of symptoms, which may have affected our findings.

## Conclusion

Overweight females with DLD exhibited compromised standing balance and functional performance. Our study found that android-type overweight individuals had excessive visceral fat, leading to spinal misalignment. This misalignment potentially contributes to postural instability and functional limitations. The findings indicate that overweight patients with DLD with an android-type body fat distribution demonstrate poorer standing balance in the AP direction compared with the other two DLD types. Moreover, the android-type overweight group showed insufficient muscle strength and muscle mass in the lower extremities. These findings have implications for physical therapists in designing interventions, as they imply that patients with DLD may benefit from specific standing balance training, functional training, and lower extremities muscle-strengthening tailored to their body fat distribution in order to achieve the best outcomes.

## Data Availability

The raw data supporting the conclusion of this article will be made available by the authors, without undue reservation.
